# Lumbar Kinematics Assessment of Patients with Chronic Low Back Pain in Three Bridge Tests Using Miniaturized Sensors

**DOI:** 10.3390/bioengineering10030339

**Published:** 2023-03-08

**Authors:** Athanasios Triantafyllou, Georgios Papagiannis, Sophia Stasi, Panagiotis Gkrilias, Maria Kyriakidou, Effrosyni Kampouroglou, Apostolos-Zacharias Skouras, Charilaos Tsolakis, George Georgoudis, Olga Savvidou, Panayiotis Papagelopoulos, Panagiotis Koulouvaris

**Affiliations:** 1 Biomechanics Laboratory, Department of Physiotherapy, University of the Peloponnese, 23100 Sparta, Greece; 2 Sports Excellence, 1st Department of Orthopaedic Surgery, National and Kapodistrian University of Athens, 12462 Athens, Greece; 3 Laboratory of Neuromuscular and Cardiovascular Study of Motion, Physiotherapy Department, Faculty of Health and Care Science, University of West Attica, 12243 Egaleo, Greece; 4 2nd Department of Surgery, Aretaieio Hospital, National and Kapodistrian University of Athens, 11528 Athens, Greece; 5 Sports Performance Laboratory, School of Physical Education & Sports Science, National and Kapodistrian University of Athens, 17237 Athens, Greece; 6 Department of Physiotherapy, University of West Attica, 12243 Athens, Greece

**Keywords:** lumbar kinematics, bridge tests, IMUs, miniaturized sensors, low back patients

## Abstract

Lumbar muscle atrophy, diminished strength, stamina, and increased fatigability have been associated with chronic nonspecific low back pain (LBP). When evaluating patients with LBP, trunk or core stability, provided by the performance and coordination of trunk muscles, appears to be essential. Several clinical tests have been developed to identify deficiencies in trunk performance, demonstrating high levels of validity and reproducibility. The most frequently prescribed tests for assessing the core body muscles are the prone plank bridge test (PBT), the side bridge test (SBT), and the supine bridge test (SUBT). However, quantitative assessments of the kinematics of the lumbar spine during their execution have not yet been conducted. The purpose of our study was to provide objective biomechanical data for the assessment of LBP patients. The lumbar spine ranges of motion of 22 healthy subjects (Group A) and 25 patients diagnosed with chronic LBP (Group B) were measured using two inertial measurement units during the execution of the PBT, SUBT, and SBT. Statistically significant differences between the two groups were found in all three tests’ kinematic patterns. This quantitative assessment of lumbar spine motion transforms the three bridge tests into an objective biomechanical diagnostic tool for LPBs that may be used to assess the efficacy of applied rehabilitation programs.

## 1. Introduction

Long-lasting low back pain (LBP) of undefined cause has been associated with muscle atrophy, decreased strength, endurance, and excessive fatigability of the lumbar muscles [1,2,3,4,5,6,7]. In addition, prospective studies suggest that lumbar muscle deconditioning may be a common risk factor for acute LBP [6,7].

Trunk or core stability quantification—the strength, endurance, and coordination of trunk muscles—with a gold-standard method has yet to be introduced [8,9]. However, that specific parameter seems important when assessing patients with LBP [10]. Furthermore, an objective determination of trunk function can provide valuable information concerning the progression of the pathology in decision making about the optimum rehabilitation program and in evaluating its outcome [11].

Various clinical tests have been proposed to help identify deficits in trunk performance, showing excellent validity and repeatability [12,13]. In addition, reduced trunk muscular endurance has been correlated with nonspecific LBP [12,14,15] and identified as a risk factor for recurrent bouts of the condition [16,17]. The most commonly referred tests for core body musculature assessment are the prone plank bridge test (PBT) for abdominal core muscles, the side bridge test (on both sides) (SBT), and the supine bridge test (SUBT) [18]. Such field tests can be easily applied in a clinical environment without specific equipment, allowing multiple patient evaluations simultaneously [16].

IMUs have been validated and proven to be accurate instruments for the objective assessment of a variety of pathological conditions, such as asymmetrical locomotion; consequently, they are gaining popularity among clinicians for everyday practice outside of the laboratory [19]. The literature supports their established reliability and validity when used to evaluate the lower back. More specifically, the PBT is a valid and reliable measure for assessing abdominal performance, showing an ICC between testing sessions of 0.915. The SUBT presents good to excellent reliability (ICC > 0.836), while the SBT presents high to very high reliability (ICC 0.74–0.96) [20,21].

Even though these tests provide significant information for assessing LBP patients, quantitative measurements of lumbar spine kinematics during their execution have yet to be performed. Miniaturized sensors (inertial measurement units—IMUs) can offer reliable kinematic data on the corresponding kinematic pattern of the aforementioned clinical tests, hence adding a significant quantitative biomechanical component to the testing [22].

Since the prone plank bridge, supine bridge, and side bridge tests are the most commonly used functional tests for assessing patients with low back pain in daily clinical practice, the purpose of this study was to observe/measure the actual kinematics of the lumbar spine for healthy and low back pain subjects. Additionally, we examined possible differences in lumbar ROMs between two groups (as correspondingly recorded for time/endurance) to quantify the lumbar kinematics during the execution of the previously described valuable clinical tools. Eventually, the previously mentioned tests could integrate the objective biomechanical behavior of the lumbar spine, a valuable clinical parameter in low back pain patient evaluation.

## 2. Materials and Methods

**Figure 1:**
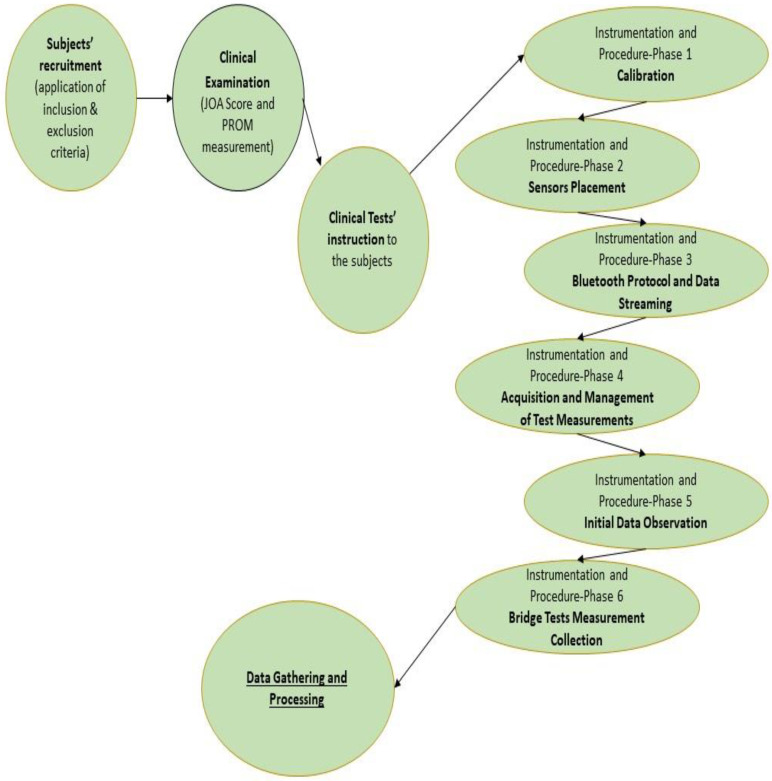
Flow Chart.

The study’s methodological approach and procedure are depicted in the following flow chart (Figure 1).

### 2.1. Subjects

A total of 53 (30 male and 23 female) patients diagnosed with chronic low back pain due to spondyloarthropathy were initially examined. The inclusion criteria were the occurrence of at least Modic type II changes in magnetic resonance imaging (MRI) and at least grade 5 interverbal disc degeneration changes according to Modified Pfirrmann grading in the absence of neurological signs. Modic changes (MC) are injury signs in the bone marrow of the vertebral body, depicted in MRI, that are related to low back pain (LBP) [23]. Similarly, the Pfirrmann classification is the most widely used categorization system for disc degenerative assessment in rehabilitation and academic settings [24,25]. Chronic low back pain due to anatomical–pathological spine alterations (spondylolysis, spondylolisthesis, scoliosis, bone fractures, neurological symptoms) and non-musculoskeletal pathological conditions such as cancer or renal disease with referral lumbar spine pain constituted the exclusion criteria. A total of 7 patients were eliminated because of sciatica, 2 because of spondylolysis, 1 because of spondylolisthesis, 3 due to radiological scoliosis abnormalities, 8 due to a history of spinal fracture, and 7 because of osteoporosis.

Finally, 22 healthy subjects (12 men and 10 women) (Group A), as well as 25 patients diagnosed with chronic low back pain (10 male and 15 female) (Group B), were included in this study. The mean age was 48.5 ± 9.3 for Group A and 49.4 ± 11.2 years for Group B (*p*-value = 0.12). The mean body mass index (BMI) was 23.3 ± 1.7 and 24.6 ± 2.4 for Groups A and B, respectively (*p*-value = 0.18). The measured parameter was the lumbar spine position, which was obtained during the execution of each of the three tests, compared to the neutral position for all subjects when they performed the PBT, the SUBT, and the SBT (Table 1).

### 2.2. Clinical Examination

Various scales for measuring physical impairment are utilized to quantify a patient’s state before and after therapy. The JOA score, created by the Japanese Orthopaedic Association in 1975, is the most frequently used measure to assess patients with low back pain. [26]. Our research evaluated both groups with a JOA score before the test execution (Table 1).

All participants’ passive flexion–extension, abduction–adduction, and left–right lumbar spine rotation were assessed prior to the clinical testing to guarantee appropriate passive ROM in all three planes and the absence of pain, hence allowing all patients to participate in the clinical tests. The passive ROMs were measured using the IMUs following the instrumentation and procedure mentioned below according to the positions described in the manual muscle test (MMT) for normal muscle strength, but the motion was executed passively by the clinician [27].

### 2.3. Clinical Tests

Prone Bridge

**Figure 2:**
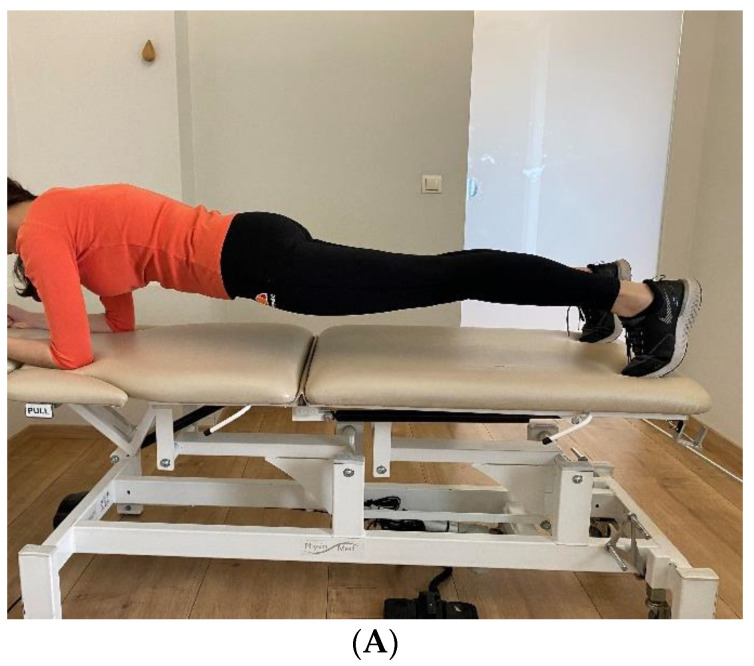

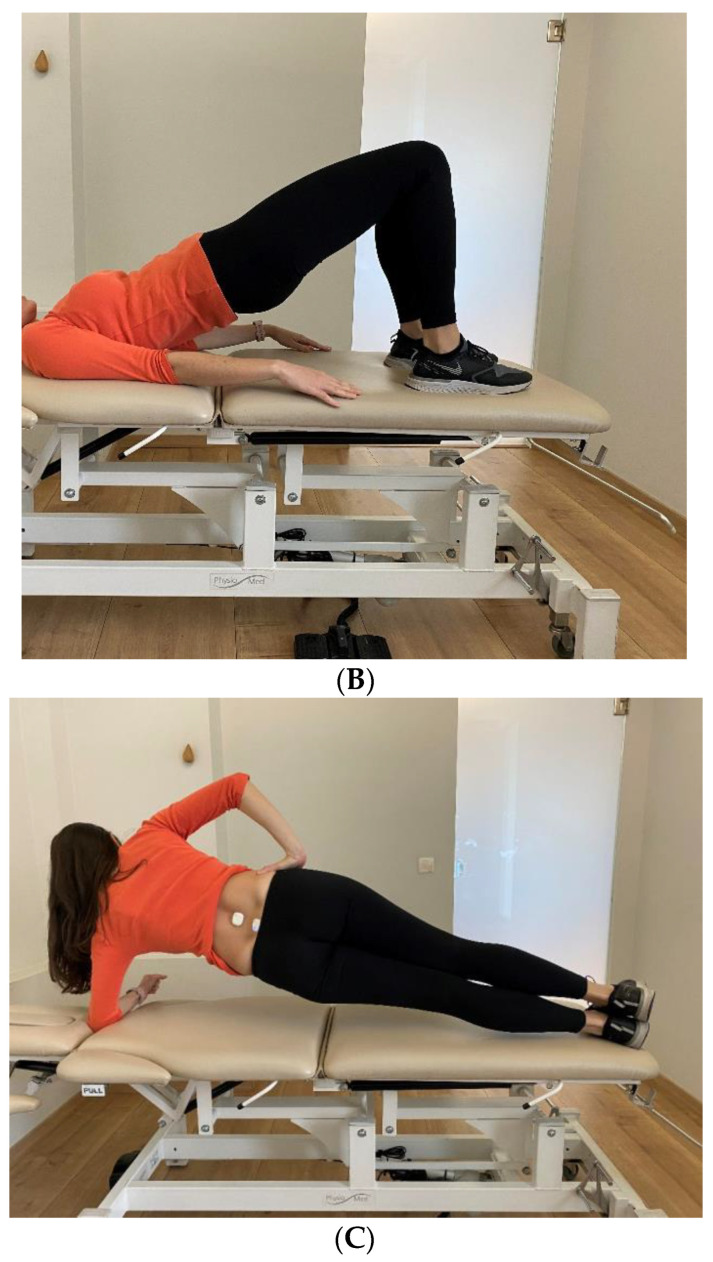
(**A**) Prone bridge test end position. (**B**) Supine bridge test end position. (**C**) Left-side bridge test end position.

Each subject was placed in the prone position on a firm surface, propped on the elbows. The elbows were separated and placed shoulder-width apart, and the feet had a narrow base but were not touching. The subject then lifted the pelvis until only the forearms and ankles touched the bed, keeping a horizontal line between the shoulders, pelvis, and ankles (Figure 2A). The test pose was maintained until fatigue or discomfort prohibited its continuation [20].

Supine Bridge

Each subject started in a supine position with their knees flexed 90 degrees and their feet on the bed close to each other but not touching. The arms were placed next to the torso. The subject lifted their pelvis from the bed, maintaining a horizontal line between the shoulders, hips, and knees (Figure 2B). The test position was sustained until fatigue or discomfort prohibited its continuation. The dominant leg was straightened in the air if the subject achieved two minutes in the test position, eliminating one support point. This was intended to reduce the duration of the bridge by adding a torque moment to the bridge’s center. In the occurrence of a previous pathology to the dominant support leg (e.g., knee pain), the opposite leg was instead moved forward [28].

Side Bridge Test

For the side bridge test, the participant was directed to recline on one side on the same examination bed. The lower supporting arm was flexed 90 degrees at the elbow, and the upper arm was folded across the torso. The subject was instructed to elevate the hips off the bed surface and keep an extended straight position with a flexed forearm directly beneath the shoulder (Figure 2C). Both sides were subjected to examination [29].

### 2.4. Instrumentation and Procedure

Throughout the prone plank bridge, supine bridge, and side bridge examinations, lumbar spine ranges of motion (ROMs) were measured. To evaluate the kinematics of the lumbar spine, two MetaMotionR+ inertial measurement units (IMUs) were utilized. MetaMotionR+ (MMR) is designed to record and stream sensor data and has CE approval. Unprocessed sensor information was recorded and transmitted at 400 Hz and 100 Hz, respectively, via Bluetooth. The researcher obtained and examined a CSV file containing data on a local PC. Data fusion integrates information from a three-dimensional gyroscopic sensor, a three-dimensional magnetometer, and a three-dimensional accelerometer to produce a stable constant direction coordinate expressed as Quaternion or Euler angles. Moreover, sophisticated software effectively integrates the unprocessed instrument data to improve each instrument’s performance. This method includes techniques for offset correction of each sensor, standardization status monitoring, and Kalman filter integration to produce corrected and usable orientation coordinates. MMR+ is an established device for recording human motion data and monitoring gestures. A list of its technological components is as follows:

- BMI160 6-Axis Accelerometer + Gyroscope;
- LTR-329ALS Luminosity/Ambient Light;
- BMM150 3-Axis Magnetometer;
- BOSCH 9-Axis Sensor Fusion;
- 8 MB memory;
- Lithium-ion rechargeable battery;
- Vibrating coin motor;
- Low-energy Bluetooth, CPU, button, LED, and GPIOs.

A Bluetooth radio embedded in each IMU delivers data wirelessly to a researcher’s smartphone. IMUs and Bluetooth radios are battery-powered with 3.6 V cells. The data collection procedure comprises six distinct phases.

#### 2.4.1. Phase One—Calibration

For human movement assessment using an inertial sensing system, it is necessary to determine the instrument reference and body orientation frame correspondence [21]. Prior to attaching and activating the sensors, the IMUs were adjusted on a flat, horizontal plane to assure that each unit had the same initial position as the coordinate system. Therefore, we assumed that the first and fifth lumbar vertebral segments are in the same plane. This enables the collection of measurements with the lumbar section coordinated with a designated axis of the global reference. It is vital to adhere to a reliable calibration process to acquire reliable data for motion assessment. The calibration was conducted on a smartphone running the Metawear application developed by mbientlab INC (software version: 2.0.1).

#### 2.4.2. Phase Two—Sensor Placement

**Figure 3:**
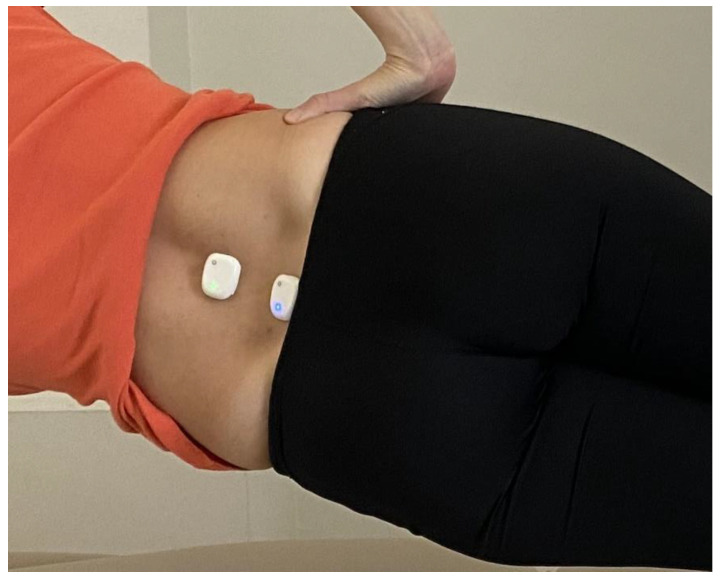
IMUs’ placement on L1 and L5 vertebrae.

The positioning of the sensors was carefully carried out to precisely determine the respective orientation and location of the device’s frame in relation to the plane of physical motion [30,31]. The first IMU was mounted directly above the spinous process of the first lumbar vertebra, while the second was placed directly above the spinous process of the fifth lumbar vertebra. Using hypoallergenic double-sided adhesive, both sensors were adhered to the epidermis (Figure 3).

#### 2.4.3. Phase Three—Bluetooth Protocol and Data Streaming

These IMU sensors interacted via Bluetooth with a smartphone. Metawear, mbientlab INC’s application (software version: 2.0.1), was loaded on the smartphone to capture and save data. After each session, the collected data were streamed over Bluetooth to a PC for further analysis. The analysis included pre- and post-filtering of three-axis acceleration, geomagnetic tracking and alignment (roll, pitch, and yaw), and three-dimensional angular acceleration in the local sensor coordinate system.

#### 2.4.4. Phase Four—Acquisition and Management of Test Measurements

Before collecting clinical test data, the same clinician performed passive lumbosacral section flexion–extension and left–right lateral flexion to validate the method’s repeatability and ensure that both wearable sensors performed as intended and acquired ROM data. Extension passive range of motion (PROM) was measured with the patient lying in the prone position on an examination bed. A special belt stabilized the patient’s pelvis at the bed, and one examiner stabilized the same anatomical region with his hands. At this position, the IMU data showed 0 degrees of ROM. A second examiner wrapped his hands around the subject’s chest and executed lumbar spine extension till he felt a firm “end feel” and no more motion could be produced. The “End Feel” is a type of sensation or feeling that examiners experience when the joint is at the end of its available passive range of motion in assessment [32]. At that point, the overall passive lumbar extension ROM was obtained.

Flexion and lateral flexion PROMs were examined with the patient lying in a supine position on an examination bed. A special belt stabilized the patient’s pelvis at the bed, and one examiner stabilized the same anatomical region with his hands. At this position, the IMU data showed 0 degrees of ROM. The second examiner wrapped his hands around the subject’s thoracic spine and executed lumbar spine flexion until he felt a firm “end feel” and no more motion could be produced. At that point, the overall passive flexion ROM was obtained. As for the right and left lateral flexion, the same methodology was applied regarding pelvis stabilization and the IMU’s reference position of 0 degrees. Following this, the second examiner wrapped his hands around the subject’s thoracic spine and executed lumbar spine lateral flexion (first to the left, then to the right) till he felt a firm “end feel” and no more motion could be produced. At that point, the overall passive lateral flexion ROM was obtained for both sides.

These records were saved as a CSV file and transmitted directly to the PC’s local storage (one for each sensor). All subjects were instructed and prepped for the technique described above. This experiment was performed three times for each ROM to acquire representative motion data.

#### 2.4.5. Phase Five—Initial Material Observation

Even if the wearables’ precision is high and the root mean square error (RMS) is employed, a computerized body representation enables the researcher to determine whether movement reconstruction and data acquisition were managed effectively. After the passive ROM measurement experiment was completed, the collected data were examined for irrelevant outcomes. If errors or improper motions were detected due to magnetic field interference, the calibration was examined to eliminate the disturbance while the participants were still present so the measurement could be repeated if necessary.

#### 2.4.6. Phase Six—Bridge Test Measurement Collection

After a successful test session, the patients were asked to perform the prone plank, supine bridge, and side bridge positions. All patients were given clear instructions about the procedure, and they performed two trials for familiarization before data measurements [28]. In addition, they were instructed to carry the smartphone next to them throughout the assessment to prevent signal connectivity problems during the data capture and transmission from the IMU to the smartphone. After the measurements were complete, the clinician removed the sensors from the patients and transferred the collected data from the smartphone to the PC.

### 2.5. Data Gathering and Processing

Two nine-dimensional inertial units were used to evaluate lumbar spine motion ranges. Mounted on the subject’s body, the sensors can remotely stream, via Bluetooth, a 3D vector of the gyroscope’s angular velocity (deg/s) in the three planes (x, y, z). Likewise, a 3D vector of the accelerometer and the magnetometer elements are transmitted. Combining the information collected from the three aforementioned elements of the sensors, which eliminates potential errors [33], the orientation of the body section and accompanying Euler angles may be appropriately determined.

The MMR+ NDoF fusion algorithm calibrated the sensor’s background automatically. The accelerometer, gyroscope, and magnetometer provide the combined precise position values in this 9-axis setting. The benefits of integrating all three sensors include rapid computation, leading to an increased output data stream, and enhanced resistance to geomagnetic distortion. In this mode, the option for rapid magnetometer calibration was enabled, resulting in more precise output values. NDoF option implemented the following frequency content: The fusion technique uses the accelerometer sensor’s output to adjust for gyroscope disturbance and measurement variations. When the subject’s target section is in motion, the fusion promptly dismisses accelerometer sensor readings in favor of gyroscope data for pitch and roll. If accelerometer readings are ignored for a prolonged time, pitch and roll values may fluctuate (due to continual movement or vibration). The same will happen if the fluctuation affects the magnetic measurements; the program will, by default, overlook it.

Let x = [a x, a y, a z, g x, g y, g z, m x, m y, m z] correspond to one inertial unit’s measurement. Calculating the Euler angles using the x data is the initial stage. Then, the L1 and L5 direction vectors are calculated using information from all three elements (accelerometer, gyroscope, magnetometer). Therefore, the variables a x, g x, and m x are converted to Euler angles of the pitch plane since they correspond to the lumbar region ROM angles [34]. To this end, the method proposed by Qifan Zhou et al. 2021 [35] was implemented, allowing a Kalman filter to determine the sensor’s exact direction [36]. The Kalman filter is a system used to depict nonlinear systems ruled by nonlinear differential equations. The IMU data are provided during the update phase, and after four defined steps based on the filter components are completed, the outcome is a position prediction [34,36]. Lastly, typical Euler angles [36] were employed to compute the lower back ROM angles relative to the L1-L5 coordinate system using a 5 Hz data frequency.

## 3. Results

The independent t-test, one-tailed distribution, and two sample unequal variance were used for the data statistical analysis. The statistical power of our study was calculated using G*Power 3.1.9.7 software for Windows (Heinrich-Heine-Universität Düsseldorf, Düsseldorf, Germany). The analysis was conducted for each dependent variable, which tested lumbar spine kinematics during the execution of the three performed tests. A power analysis for two independent means was employed with a given alpha = 0.05. The power of the study’s sample size and observed effect size was higher than 80% for each variable (96.5% for the prone bridge test, 88.2% for the left-side bridge test, close to its maximum of 1 for the supine bridge test, and the right-side bridge test). An intraclass correlation coefficient (ICC; two-way random, absolute agreement) and respective 95% confidence interval were used for relative reliability analysis and to interpret results for within-session measurements in all bridge tests and passive ROM clinical examinations (ICC > 0.90).

### 3.1. Clinical Examination

#### 3.1.1. Passive Range of Motion

No statistically significant differences were found regarding the passive ROM of flexion (Group A: mean = 49.33°, SD = 0.94 and Group B: mean = 48.83°, SD = 1.12, *p*-value = 0.24), the passive ROM of extension (Group A: mean = 14.66°, SD = 0.94 and Group B: mean = 14.16°, SD = 0.68, *p*-value = 0.18), the passive ROM of right lateral flexion (Group A: mean = 18.67°, SD = 0.74 and Group B: mean = 17.83°, SD = 1.06, *p*-value = 0.09), or the left lateral flexion (Group A: mean = 18.34°, SD= 0.74 and Group B: mean = 18.5°, SD = 0.95, *p*-value = 0.38) (Table 2).

#### 3.1.2. The Japanese Orthopaedic Association (JOA) Score

The JOA score was used to analyze leg pain, low back pain, and everyday activities in both groups, healthy and LBP patients. This method has a maximum score of 29 points, and higher JOA scores indicate better outcomes for the analyzed criteria [37]. Statistically significant differences were found between the two groups (*p*-value = 0.0003). The average healthy subjects’ JOA score was 27.15 (SD) = 0.73) (Group A), while the corresponding values for LBP patients were evaluated at 22.2 (SD = 1.01) (Group B).

### 3.2. Wearable Sensors—IMU Data

All measured tests presented statistically significant differences between the examined groups, as the *p*-values mentioned in each case indicate.

Supine Bridge Test

**Figure 4:**
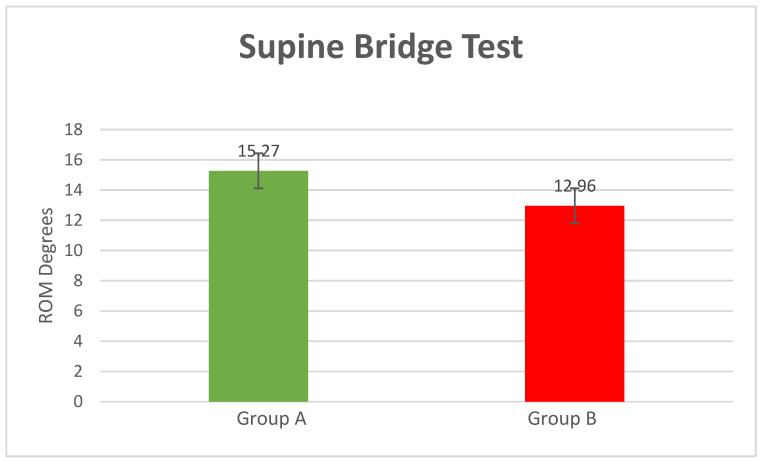
Supine bridge test results (*p*-value = 0.00004).

The position obtained during the execution of the supine bridge test was extension (compared to the neutral position), with patients m: mean = 15.27° of extension SD = 1.42 for Group A and mean = 12.96° of extension SD = 2.10 for Group B (*p*-value = 0.00004) (Figure 4).

Prone Bridge Test

**Figure 5:**
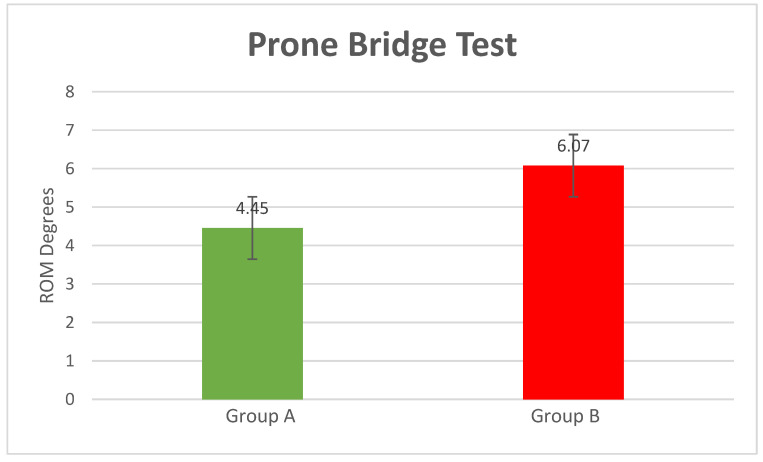
Prone bridge test results (*p*-value = 0.0001).

The position obtained during the execution of the prone bridge test was extension (compared to the neutral position), with the patients measured at: mean = 4.45° of extension SD = 1.07 for Group A and mean = 6.07° of extension SD = 1.97 for Group B (*p*-value = 0.0001) (Figure 5).

Left-Side Bridge Test

**Figure 6:**
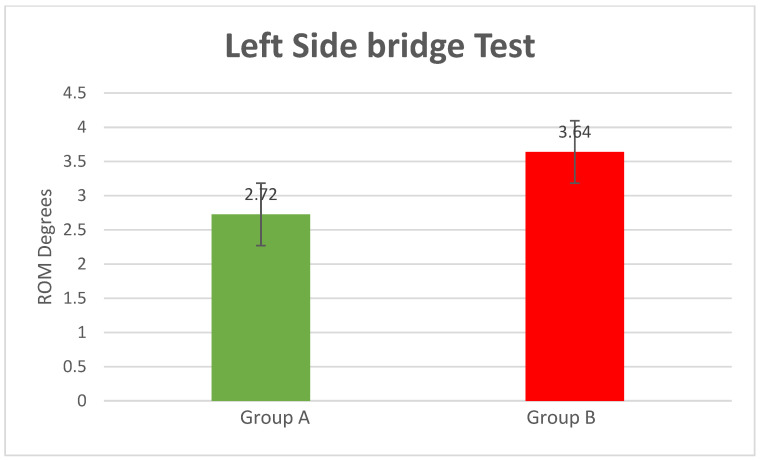
Left-side bridge test results (*p*-value = 0.0004).

The position obtained during the execution of the left-side bridge test was right lateral flexion (compared to the neutral position), with patients achieving the following values: mean = 2.72° of right lateral flexion SD = 1.10 for Group A and mean = 3.64° of right lateral flexion SD = 1.09 for Group B (*p*-value = 0.0004) (Figure 6).

Right-Side Bridge Test

**Figure 7:**
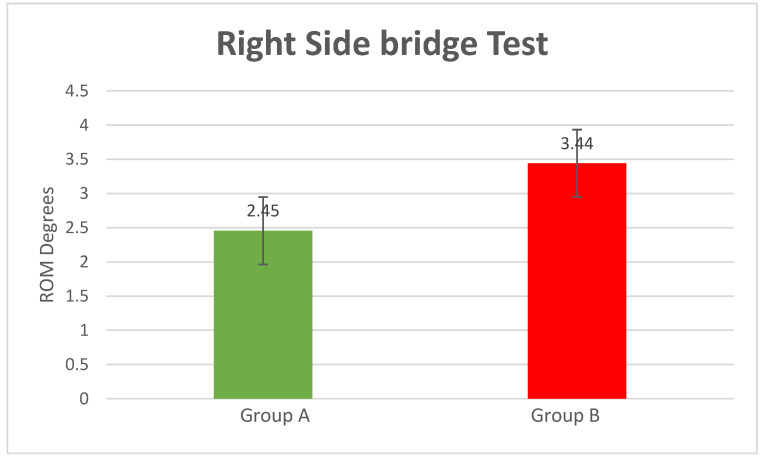
Right-side bridge test results (*p*-value = 0.00002).

The position obtained during the execution of the right-side bridge test was left lateral flexion (compared to the neutral position), with the patients measured at: mean = 2.45° of left lateral flexion SD = 0.58 for Group A and mean = 3.44° of left lateral flexion SD = 0.85 for Group B (*p*-value = 0.00002) (Figure 7).

**Figure 8:**
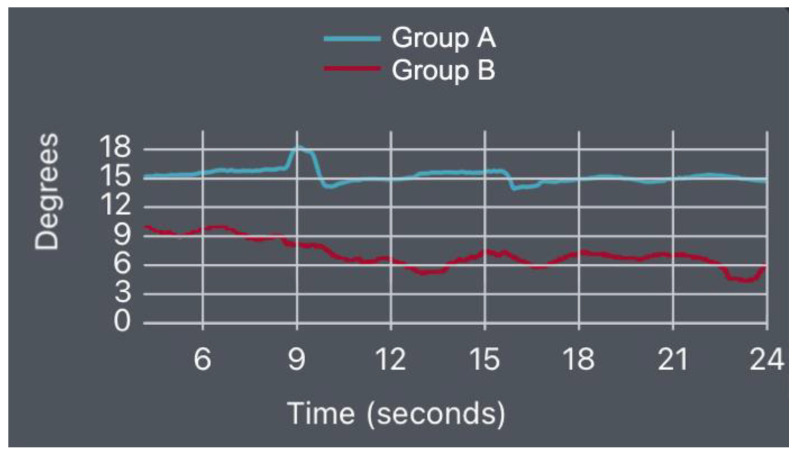
SUBT flexion–extension diagram for Groups A and B.

**Figure 9:**
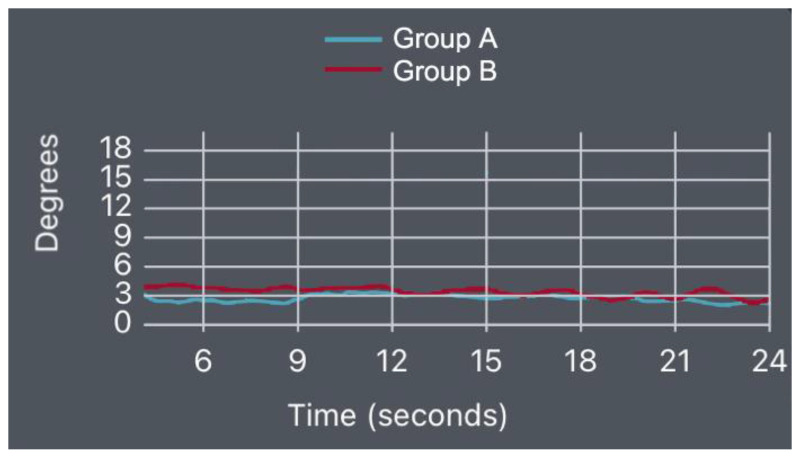
PBT flexion–extension diagram for Groups A and B.

**Figure 10:**
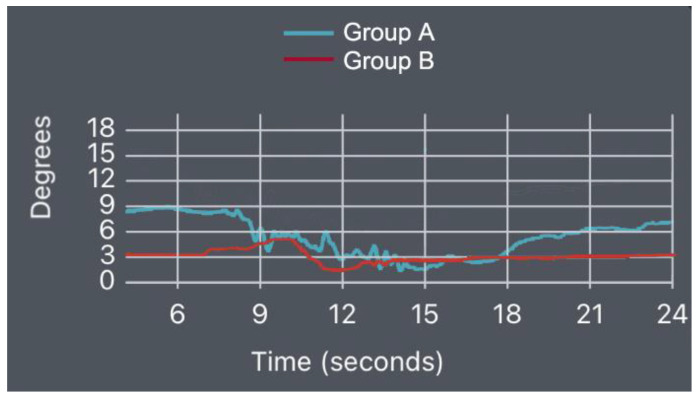
Left-SBT lateral flexion diagram for Groups A and B.

We present some indicative diagrams for the lumbar spine kinematics behavior for both groups in the three tests, as shown in Figure 8, Figure 9 and Figure 10.

## 4. Discussion

The clinical assessment of patients with lumbar spine dysfunction includes testing for an abnormal gross range of motion at the three axes of motion [38]. Prone plank bridge, supine bridge, and side bridge tests are the most commonly used functional tests for assessing patients with low back problems in daily clinical practice. However, they do not evaluate possible kinematic impairments for LBP patients compared to healthy individuals [39,40]. In our study, we introduced and measured for the first time, to our knowledge, the kinematic components of the most widely used clinical tests for low back pain assessment. The measured parameter was the lumbar spine position obtained during the execution of each of the three tests compared to the neutral position. The supine bridge test evaluated lumbar extension, the prone bridge test evaluated lumbar flexion, the left-side bridge test evaluated right lateral flexion, and the right-side bridge test evaluated left lateral flexion. We identified statistically significant differences for all measured tests between the examined groups.

Researchers report significant losses in the range of lumbar spine movement in LBP subjects in all directions, for example, loss of hip flexion and altered hip and lumbar spine kinematics while performing trunk movements [41] and between LBP subgroups, some of which involve the action of lumbar spine extension [42,43]. Their results agree with our data when kinematics are measured through clinical tests.

Despite the lack of information about the kinematics of the lumbar spine during these three bridge tests, a number of other experiments examining lumbar spine ROMs during the execution of comparable exercises have been described. Milosavljevic et al. (2008) report mean peak values of angular displacement of the lumbar spine at 17.5 ± 5.6 degrees when their healthy young subjects performed trunk bending backward and returning to the upright position [38]. These values for peak angular displacement for the lumbar spine and hip are also consistent with those obtained in other studies [41,44]. Our study measured the mean peak values of angular displacement at the supine bridge test for Group A (healthy subjects) at 15.27 degrees.

Our results show less ROM in LBP patients compared to healthy individuals and a shorter duration of the test execution (the time at which the test stopped because of pain). This might suggest that the statistically significant differences found in the ROMs in all three planes and the reduced duration for which patients could maintain the test position could result from muscle strength deficits (since all subjects had a normal passive ROM and could potentially perform similar active ROM scores). Thus, a kinesiotherapy exercise program targeting these muscle groups to increase their strength and endurance could help LBP patients achieve better active ROMs and time scores for our tests and, as a result, reduce symptoms and improve functionality [45,46,47,48,49].

Vanti et al. l [50] discovered an improvement in results in symptomatic lumbar spondylolisthesis patients prior to and following treatment (time duration). In addition, they demonstrated the sensitivity of bridge tests since their results were substantially correlated with discomfort and functional changes. Changes in bridge test scores also appeared to be associated with the degree of perceived progress as evaluated by the Global Perceived Effect questionnaire and were consistent with other quality indicators (Oswestry Disability Index—Italian version and the Pain Numerical Rating Scale). In the literature, bridge movements have primarily been investigated as a treatment technique to increase muscle strength and performance of the lower back area in patients with nonspecific LBP [51,52,53], progressing from reduced-intensity exercises to high-intensity [54].

In the past, physicians evaluated a patient’s success in bridge tests (BTs) based on the length of time the patient could hold the relevant posture. With our study, we emphasize the need to correlate these findings with kinematic data to determine whether the improvement observed after rehabilitation protocols also reflects improvement in the functional capacity (kinematics improvement) of these patients, which is a crucial factor in treatment decision making. This quantitative assessment of lumbar spine motion in all three planes converts the prone plank test, supine bridge test, and side bridge test into an objective biomechanical diagnostic tool for LPBs that may be used to evaluate the success of applied rehabilitation programs. However, no study has yet been conducted on the response to the clinical change (kinematics) over time of these tests in patients with LBP. In clinical practice, the clinician evaluates the patients subjectively by visually controlling the correct posture but is not involved in any way in helping patients maintain the position during the test execution [50]. Our study introduces an objective component for the assessment of LBP patients by measuring kinematic parameters, which also reflect the functional ability of LPB patients. Thus, BTs can additionally be utilized to identify clinical and quantitative shifts in chronic LBP patients following rehabilitation treatment. In addition, information on kinematics may enable the physiotherapist to determine the importance of a variation in BTs between the reference point (typically the first session) and each successive phase of treatment. Therefore, additional research should be conducted on using BTs during and after the completion of a physical therapy program for chronic LBP, and not only as an assessment tool to evaluate the fitness level of the core lower back musculature. Furthermore, the advancement in BTs scores, in lumbar kinematics, and the overall improvement in low back discomfort and disability, is significantly clinically relevant, as these easy tests that can be conducted daily in a clinical environment, can assist rehabilitation professionals in objectively evaluating their patients’ improvement.

## 5. Limitations

Conventional motion capture with reflector skin markers is widely used to quantify joint kinematics; nevertheless, the relative movement occurring at the biological tissue between the bones and markers produces a source of measurement error called soft tissue artifacts (STAs). STAs are also prevalent when IMUs are attached to the skin of the lumbar spine to capture the lumbar spine’s range of motion. Xin HI et al. employed dual fluoroscopic techniques to assess STAs in lumbar spine motion in healthy adult participants. During extension, they discovered that the lower lumbar region (L4–L5: 2.7 ± 0.7 mm) had considerably fewer STAs than the upper lumbar region (L1–L3: 6.1 ± 3.3 mm) [55]. Likewise, Zemp et al. report a mean STA of 10.7 ± 4.8 mm in lumbar extended postures when comparing skin marker motion to MRI images [56]. Given the fact that IMUs are mounted to the skin in the same manner as skin markers of optoelectronic motion capture systems and that lumbar spinous process mean height dimensions vary from 18 to 27 mm in adults [57], it appears that even though STAs occur and present a potential source of inaccuracy in such measurements, the surface equipment’s skin position (markers and IMUs) should still coincide with the underlying spinous process when a full range of motion is executed.

In an effort to further examine the phenomenon in which the motion of the skin does not reflect the motion of the underlying vertebrae perfectly, W.K. Chiou et al. aimed to establish a relationship between skin surface motion and intervertebral motion [58]. The researchers computed and compared the intersegmental joint angles of the lumbar spine during sagittal plane motion using a digitized and transformed data model. In their investigation, the non-invasive and invasive ISA differences varied from 0.29 to 2.08 degrees of the complete range of flexion ROM (0.58 to 5.6% of the full ROM).

Consistent with previous results, Yang et al. report estimation errors of surface-attached sensors in monitoring flexion movement of the lumbar region of approximately 5% of the full range of motion compared to those attained with computed tomography in capturing the entire lumbar spine position (used as the “gold” standard), therefore concluding that kinematic measurements with skin-mounted sensors are sufficiently accurate [59].

Similarly, Ha, Tshui-Hung, and colleagues sought to validate the use of IMUs in measuring the three-dimensional spinal range of motion using an electromagnetic tracking system as a benchmark. They discovered that measurements acquired with the inertial measuring system were strongly correlated with the electromagnetic tracking system, exhibited great agreement, and did not have any major differences from the electromagnetic tracking system. Thus, they concluded that inertial measurement systems containing a gyroscope, an accelerometer, and magnetometer sensors (IMUs with these components were used in our study) are accurate instruments for assessing the three-dimensional spinal range of motion and that their cost-effectiveness, compact footprint, and ease of use offer significant advantages in such measurements [60].

Leroy reports that soft tissue and skin movement can induce IMUs to change their positions relative to the body’s bony prominence. However, these artifacts’ impact is minor during low-intensity activities (such as the three-bridge test assessed in our study) [61].

## 6. Conclusions

The development of miniature sensors has created new opportunities for simultaneous and long-term recording and the quantification of human body kinematics, including objective evaluation of motor abnormalities that spontaneously emerge and are recreated by individuals in real-world settings [62,63]. Their lightness, compact size, relatively inexpensive cost, energy efficiency, and mobility characteristics make them appropriate for several applications, providing an objective assessment component for various clinical tests. Spatiotemporal parameters (angular velocity, acceleration) that can also be recorded with IMUs could provide more detailed information about muscular performance, and thus further research in that direction should be promoted.

## Figures and Tables

**Table 1:** Demographic data and JOA scores.

Item		Healthy (Mean/±SD)	Low Back Pain (Mean/±SD)	*p*-Value
Total Number		22	25	
Gender	Male	12	10	
	Female	10	15	
Age		48.5/±9.3	49.4/±11.2	0.12
BMI		23.3/±1.7	24.6/±2.4	0.18
JOA Score		27.15/±0.73	22.2/±1.01	0.0003

**Table 2:** Passive range of motion measurements in Group A and Group B.

Item	Group A (Mean/±SD)	Group B (Mean/±SD)	*p*-Value
Lumbar flexion	49.33°/±0.94	48.83°/±1.12	0.24
Lumbar extension	14.66°/±0.94	14.16°/±0.68	0.18
Lumbar right lateral flexion	18.67°/±0.74	17.83°/±1.06	0.09
Lumbar left lateral flexion	18.34°/±0.74	18.05°/±0.95	0.38

## Data Availability

All data supporting the study’s reported results are stored in the local computer data network of the Biomechanics Laboratory Department of Physiotherapy, University of the Peloponnese, and are available on request from the corresponding author. The data are not publicly available due to the Laboratory’s personal data privacy policy.
